# A Very Proper Thing

**Published:** 1898-10

**Authors:** 


### A Very Proper Thing.

At the recent meeting of the National Dental Association in the selection of officers, Dr. G. H. Cushing was selected to succeed himself as secretary of that body. Dr. Cushing's health has been failing for two or three years and a few months ago he found it necessary to go to California with the hope of recuperating, and there is some hope that he may realize his desire in this respect.

The Association sharing in this hope and not being willing to at once sever Dr. Cushing's connection with this body retained him as its secretary, the duties of which office Dr. Cushing has discharged in an eminently satisfactory way during the last twenty years.

The selection of Dr. Cushing for this position was by the unanimous voice of the body, and the hope and desire is that he may be again brought to health and return to take up the active duties of the position which he so long and so honorably filled, professional and otherwise.

